# Resistance to *Haemonchus contortus* in Corriedale sheep is associated to high parasite-specific IgA titer and a systemic Th2 immune response

**DOI:** 10.1038/s41598-019-55447-6

**Published:** 2019-12-20

**Authors:** Cecilia Escribano, Anderson Saravia, Monique Costa, Daniel Castells, Gabriel Ciappesoni, Franklin Riet-Correa, Teresa Freire

**Affiliations:** 1 Grupo de Inmunomodulación y Desarrollo de Vacunas, Departamento de Inmunobiología, Facultad de Medicina, Universidad de La República, Montevideo, Uruguay; 2 Plataforma de Salud Animal, Instituto Nacional de Investigación Agropecuaria, La Estanzuela, Uruguay; 3 Secretariado Uruguayo de Lana, Florida, Uruguay; 4 Programa Carne y Lana. Instituto Nacional de Investigación Agropecuaria, Las Brujas, Uruguay

**Keywords:** Cellular immunity, Humoral immunity

## Abstract

Gastrointestinal nematode infections, including *Haemonchus contortus*, are one of the main causes of economic losses to ovine farmers worldwide. In order to contribute to the control of nematode infections and avoid parasite spreading we generated divergent resistant and susceptible sheep breeds and evaluated the adaptive immunity of these animals developed upon experimental infection against *H. contortus*. The selection of resistant or susceptible animals from the Corriedale Breed has been based on Expected Progeny Differences for faecal egg counts per gram. Furthermore, animals from the resistant Corriedale line were inseminated with imported semen from Australian Rylington Merino rams. Thus, the objective of this work was to analyze the adaptive immune response in both susceptible and resistant obtained lambs. Our results indicate that there is a potent parasite-specific local and systemic immune response in resistant animals and that although susceptible lambs can produce high levels of IgA antibodies during the infection, their antibody response is delayed which, together with an impaired specific-Th2 response, does not contribute to initial parasite elimination. Our results shed light into the immune mechanisms that mediate resistance to *H. contortus* and could constitute important assets to sheep farmers, not only as a means to detect resistance, but also to enhance the efficiency of selection in stud flocks.

## Introduction

Gastrointestinal nematode infections are one of the main causes of economic losses to ovine farmers worldwide. Among nematodes, *Haemonchus contortus* is considered one of the most pathogenic and most economically important infectious agents^1^. The parasite, a hematophagous trichostrongyle nematode, causes severe anaemia, hypoproteinemia and submandibular edema in infected sheep that can lead to death depending on the parasite load^2^. Furthermore, control of nematodes by anti-helminthic is becoming increasingly difficult due to the development of parasite resistance^3^. Taking these considerations into account, the use of sheep breeds and animals within the breeds resistant to gastrointestinal nematodes is an interesting alternative strategy that has emerged to limit helminth infections and parasite spreading^2^.

In this context, from 1998, the Uruguayan Wool Secretariat initiated a genetic program to develop divergent lines for resistance to nematodes in Corriedale breed. The selection of resistant or susceptible (R/S) animals has been based on Expected Progeny Differences (EPD) for faecal egg counts per gram (EPG), as an indicator of the genetic merit of R/S to nematodes^4^. Furthermore, animals from the resistant Corriedale line were inseminated with imported semen from Australian Merino rams. These rams come from the Rylington Merino internal parasite resistant selection line initiated in 1987 in Australia^5^.

Different independent works have established that resistance to gastrointestinal nematodes is largely governed by immunological responses directed towards larval stages of the parasites, and that highly depends on the type of the elicited immunity. Indeed, resistance to *H. contortus* is associated with potent polarized Th2 responses that are characterized by the presence of eosinophils, mast cells, and antibody production at the site of infection, as well as the production of type-2 cytokines such as IL-4, IL-5 and IL-13 in abomasal tissues or draining lymph nodes^6–9^. Finally, resistant sheep may also present higher levels of circulating antibodies than susceptible breeds^10^.

Aiming to better understand the mechanisms involved in host resistance against gastrointestinal nematode infections, in this work we evaluated the resistance/susceptibility of the previously mentioned sheep lines to *H. contortus* infection as well as the parasite-specific humoral and cellular immunity. To this end, we analyzed specific antibody titres in saliva and plasma as well as determined type-2 cytokines specifically produced by PBMC upon parasite stimuli. Our results indicate that there is a potent parasite-specific local and systemic immune response in resistant animals and that although susceptible lambs can produce high levels of IgA antibodies during the infection, their antibody response is delayed which, together with an impaired specific-Th2 response, does not contribute to initial parasite elimination. Our results shed light into the immune mechanisms that mediate resistance to *H. contortus* and could constitute important assets to sheep farmers, not only as a means to detect resistance, but also to enhance the efficiency of selection in stud flocks.

## Results

### Sheep from both Corriedale and Rylington x Corriedale lines showed high resistance to *H. contortus* infection

**Figure 1:**
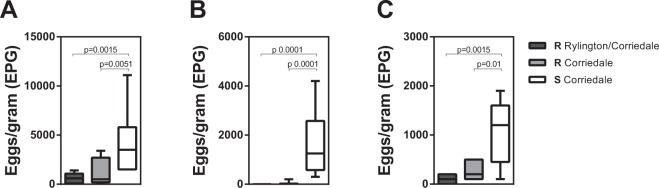
Resistant sheep lines present lower faecal EPG levels than the susceptible Corriedale line in natural infections. Faecal EPG obtained from selected resistant (R) and susceptible (S) Corriedale (n = 12) and resistant Rylington x Corriedale (n = 8) lambs during three independent periods (**A**–**C**) of natural infection of gastrointestinal nematodes.

**Figure 2:**
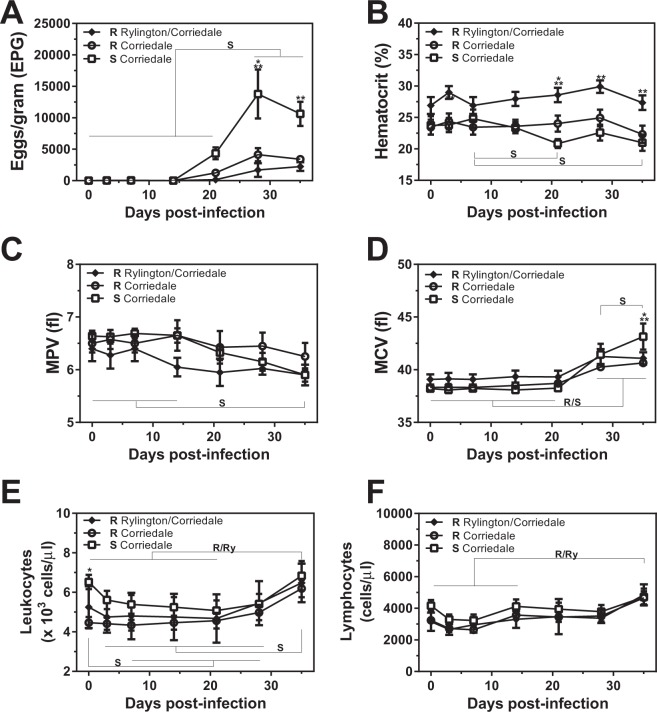
Resistant sheep lines present lower faecal EPG levels than the susceptible Corriedale line upon experimental infection with *H. contortus*. Resistant (R) and susceptible (S) Corriedale (n = 8) and resistant Rylington x Corriedale (n = 6) lambs were orally inoculated with 7500 infective L3 of *H. contortus*. (**A**) Faecal EPG of infected lambs determined by the modified McMaster technique. Hemotocrit (**B**), MPV (**C**) and MCV (**D**) of infected animals were determined using an automated Counter. Blood smears were prepared on individually labelled microscope slides and stained with Giemsa to analyze leukocyte (**E**) and lymphocyte (**F**) counts. Asterisks indicate statistically significant differences (p < 0.05) between resistant Corriedale and susceptible Corriedale lines (*) or between resistant Rylington x Corriedale and susceptible Corriedale lines (**). Bars indicate significant differences upon time of the infection for each group of animals (R, Resistant Corriedale, Ry, Resistant Rylington x Corriedale, S, Susceptible Corriedale).

In the current study we used two Corriedale sheep lines with different predicted genetic susceptibility to gastrointestinal nematodes, and selected them according to their resistance or susceptibility of infection. We also compared a Rylington Merino x Corriedale crossbreed (referred here as Rylington x Corriedale) that demonstrated, upon selection, higher resistance to natural infection to nematodes in different independent periods (Fig. 1). Furthermore, after experimental infection, lambs from the susceptible Corriedale line showed significant higher levels of faecal EPG after 28 days post-infection (dpi) than the two resistant lines (Fig. 2A). Interestingly, the increment in faecal EPG was due to a sustained significant increase in faecal EPG in susceptible animals since both resistant lines did not show significant increase in faecal EPG during the infection period (Fig. 2A). As expected, non-infected animals, did not display significant EPG during the whole experiment (Supplementary Fig. S1).

While hematocrit remained constant during the infection in animals from both resistant lines, it significantly decreased at the end of the experiment in susceptible animals (Fig. 2B). Moreover, susceptible infected animals showed lower hematocrit levels at day 21 with respect to Corriedale resistant lambs, and at days 3, 21, 28 and 35 with respect to Rylington x Corriedale resistant animals (Fig. 2B), indicating an agreement with higher EPG levels. In addition, the resistant animals from the Rylington x Corriedale crossbreed showed significantly higher hematocrit levels than resistant and susceptible Corriedale lambs from day 14 after infection (Fig. 2B), which was accompanied by lower faecal EPG (Fig. 2A).

Although Mean Platelet Volume (MPV) remained constant during the infection in the two resistant lines of lambs, it decreased in susceptible lambs at day 35 as compared to days 0 to 14 (Fig. 2C). On the contrary, Mean Corpuscular Volume (MCV) increased after day 28 in the three lines, although only susceptible lambs increased MCV between days 28 and 35, resulting in significant higher MCV levels at day 35 with respect to both resistant animals (Fig. 2D). These results are in agreement with high levels of EPG in the susceptible animals.

Lambs presented similar levels of circulating leukocytes during the infection, although, interestingly, only resistant animals from Corriedale and Rylington x Corriedale lines showed higher levels of leukocytes in blood at the end of the experiment (day 35) than at the beginning (day 0) (Fig. 2E). In the same line, only blood lymphocytes from both resistant lines increased at day 35 with respect to the initial period of infection (Fig. 2F).

### Resistant sheep lines present high levels of parasite specific antibodies before the experimental infection

**Figure 3:**
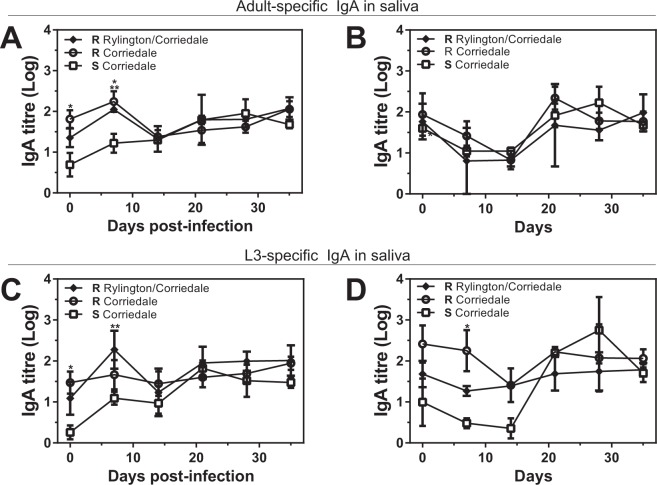
Resistant lines present higher parasite-specific IgA titres levels in saliva than susceptible lambs at the initial period of infection. Saliva samples from infected (**A**,**C**) or control (**B**,**D**) susceptible (S) and resistant (R) Corriedale and resistant Rylington x Corriedale lambs were incubated with larvae (**A**,**B**) or adult (**C**,**D**) derived-antigen coated plates. Specific antibodies were detected with anti-sheep IgA HRP-conjugated antibody. Asterisks indicate statistically significant differences (p < 0.05) between resistant Corriedale and susceptible Corriedale lines (indicated by *) or between resistant Rylington x Corriedale and susceptible Corriedale lines (indicated by **).

We next investigated the parasite-specific local and systemic humoral immune response in infected and control animals. To this end we evaluated parasite-specific IgA levels in saliva, and parasite specific-IgA and -IgG levels in plasma. Resistant infected animals from both Corriedale lines and the Rylington x Corriedale crossbreed displayed higher IgA titre levels in saliva specific to both adult and larval parasite antigens at the initial period of the infection (days 0 and 7) than susceptible Corriedale lambs (Fig. 3A,C). In addition, animals from both resistant lines showed practically constant high levels of parasite-specific IgA titres for resistant Corriedale lambs (Supplementary Fig. S2). In contrast, susceptible Corriedale lambs presented low levels of parasite-specific IgA at the beginning of the infection, and significantly augmented during the infection, reaching similar parasite-specific IgA levels in saliva than resistant animals from Corriedale and Rylington x Corriedale lines after 14 dpi (Supplementary Fig. S2). Non-infected control animals showed similar levels of adult-specific IgA in saliva (Fig. 2B and Supplementary Fig. S2). However, larval specific-IgA in saliva from susceptible Corriedale lambs were significantly lower than animals from resistant Corriedale line at day 7 (Fig. 3D) and only increased after 21 dpi (Supplementary Fig. S2).

**Figure 4:**
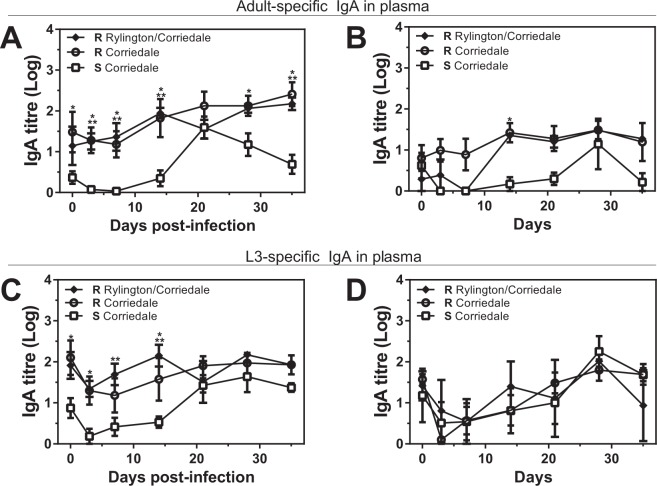
Resistant lines present higher parasite-specific IgA titres levels in plasma than susceptible lambs at the initial period of infection. Plasma samples from infected (**A**,**C**) or control (**B**,**D**) susceptible (S) and resistant (R) Corriedale and resistant Rylington x Corriedale lambs were incubated with larvae (**A**,**B**) or adult (**C**,**D**) derived-antigen coated plates. Specific antibodies were detected with anti-sheep IgA HRP-conjugated antibody. Asterisks indicate statistically significant differences (p < 0.05) between resistant Corriedale and susceptible Corriedale lines (indicated by *) or between resistant Rylington x Corriedale and susceptible Corriedale lines (indicated by **).

The delayed production of parasite-specific IgA antibodies in saliva associated to susceptibility to *H. contortus* infection was also observed, even more intensively, in plasma. Indeed, both parasite adult (Fig. 4A) and larvae- (Fig. 4C) specific IgA titres in plasma were high and constant during the total duration of the infection in both resistant Corriedale and Rylington x Corriedale lines. In contrast, susceptible Corriedale animals presented a delayed parasite-specific IgA immune response with low IgA titres at the beginning of the experimental infection, increasing only after 21 dpi (Fig. 4 and Supplementary Fig. S3). Of note, we found no difference in parasite-specific IgA titre in plasma from non infected lambs (Fig. 4B and D), except for adult-specific IgA in resistant Corriedale lambs at day 14 that was higher than susceptible Corriedale lambs (Fig. 4B). Unexpectedly, non infected animals presented an increase in parasite-specific IgA titres towards the end of the experiment (Supplementary Fig. S3), suggesting that the antibodies detected at the initial two weeks after the infection are specifically induced by experimental parasite infection and consist in the most significant difference between resistant and susceptible animals.

**Figure 5:**
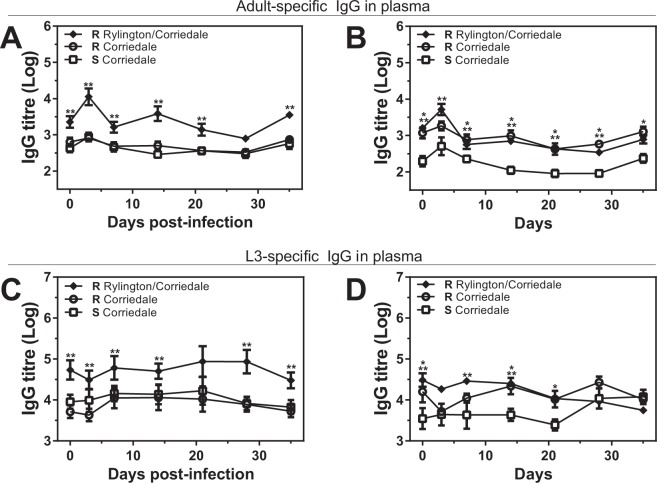
Resistant lines present higher parasite-specific IgG titres levels in plasma than susceptible lambs at the initial period of infection. Plasma samples from infected (**A**,**C**) or control (**B**,**D**) susceptible (S) and resistant (R) Corriedale and resistant Rylington x Corriedale lambs were incubated with larvae (**A**,**B**) or adult (**C**,**D**) derived-antigen coated plates. Specific antibodies were detected with anti-sheep IgG HRP-conjugated antibody. Asterisks indicate statistically significant differences (p < 0.05) between resistant Corriedale and susceptible Corriedale lines (indicated by *) or between resistant Rylington x Corriedale and susceptible Corriedale lines (indicated by **).

Finally, resistant lambs from the Rylington x Corriedale breed presented higher parasite-specific-IgG levels in plasma than resistant and susceptible Corriedale animals, both specific for adult or larval parasite-derived antigens (Fig. 5A,C). On the other hand, non infected resistant animals from both Corriedale and Rylington x Corriedale non-infected lambs had higher levels of parasite-specific IgG titres in plasma than the susceptible Corriedale group (Fig. 5B,D), suggesting that, although resistant animals from Corriedale and Rylington x Corriedale lines present high levels of IgG antibodies before infection, only the resistant animals from the Rylington x Corriedale breed have an increased capacity to produce *H. contortus*-specific IgG antibodies in plasma upon infection. Of note, no considerable changes in the IgG levels during the infections period for any of the evaluated lamb lines were detected (Supplementary Fig. S4).

### Resistance to *H. contortus* is associated with stronger type-2 cytokines produced by stimulated PBMC

**Figure 6:**
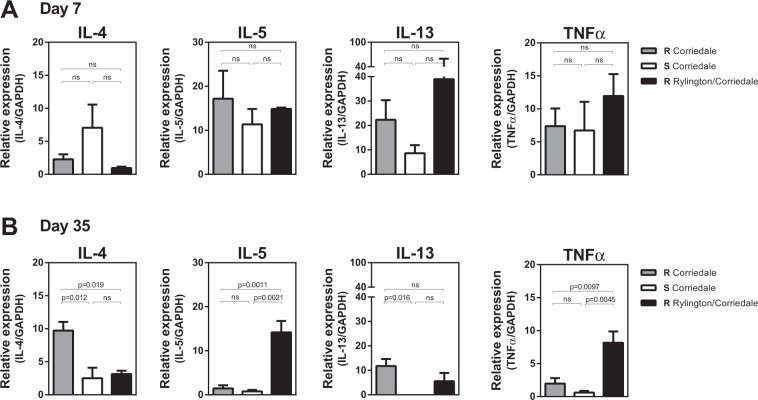
Type-2 cytokine levels produced by PBMC from resistant and susceptible lines. PBMCs from infected susceptible (S) and resistant (R) Corriedale and resistant Rylington x Corriedale lambs obtained at 7 (**A**) and 35 (**B**) days after infection were stimulated with adult-derived parasite antigens for 5 days. Copy DNA from these cells was used as a template to determine cytokine mRNA levels by qRT-PCR. Results are shown as the ratio of mRNA amplification for a specific cytokine and the GAPDH ± SEM.

Considering that type-2 immunity has been associated with resistance to infection by gastrointestinal nematodes^8,11^, we next analyzed the gene expression of classical Th2 cytokines IL-4, IL-5, IL-13. Moreover TNF*α* levels were also evaluated since it can regulate Th2 responses. To this end, we purified peripheral blood mononuclear cells (PBMC) from infected lambs and stimulated them with adult-derived antigens from *H. contortus* for 5 days. Although no differences were found in cytokine production at day 7 (Fig. 6A), PBMC from resistant Corriedale lambs produced higher levels of IL-4 and IL-13 at day 35 as compared to lambs from the susceptible Corriedale and resistant Rylington x Corriedale lines (Fig. 6B). In contrast, PBMC from resistant Rylington x Corriedale lambs produced higher levels of IL-5 and TNF*α* at day 35 as compared to lambs from the susceptible and resistant Corriedale breed (Fig. 6B). Of note, non infected animals were not able to produce significant high levels of these cytokines at day 35 (Supplementary Fig. S5). Taken together, these results indicate that resistance to *H. contortus* infection is associated to the induction of stronger Th2 cellular immune response, although different immune mechanisms were detected in both resistant lines.

## Discussion

In this work we analyzed the resistance and specific immunity induced upon experimental infection by the helminth *H. contortus* in different lines of sheep genetically selected that differ in their susceptibility/resistance to infection by gastrointestinal nematodes. Previous studies on different breed sheep have suggested that resistance is governed by an adaptive immune response that controls parasite colonization and egg production^12^. In our work we found that resistance was associated with a high parasite-specific IgA titres present in saliva since the beginning of the infection, and remained constant during the experiment, while susceptible animals had low parasite-specific IgA levels at the beginning of the study and presented increased IgA levels only at 3 weeks after infection, indicating that potency and duration of the elicited adaptive immunity, and in particular, IgA antibody levels, are key to assure resistance and parasite clearance. Furthermore, the high levels of parasite-specific IgA correlated with an increase in lymphocytes in blood in both resistant lines with respect to the initial period of infection, suggesting a more intense circulating immune response in both resistant Corriedale and Rylington x Corriedale lambs. IgA is commonly found in high levels in surface secretions, saliva, intestinal fluid, milk and colostrums^13^, and can provide protection against gastrointestinal parasites through several mechanism^14^ including larvae immobilization^15^, complement activation^16^, suppressing egg production in the adult female^17^ or by mediating parasite killing via Fc receptor binding on effector cells such as eosinophils and macrophages^14^, a process known as antibody dependent cell cytotoxicity (ADCC). Thus, we could hypothesize that IgA in saliva can recognize and bind infective *H. contortus* larvae in the mouth and gut of lambs, and then act together with mast cells or eosinophils once they reach the gut, limiting larvae number, maturation and egg shedding. Indeed, IgA specific to *Teladorsagia circumcincta* larvae mediates suppression of parasite growth, development and fecundity^18–21^.

Interestingly, parasite-specific IgA was increased not only in saliva, but also in plasma, indicating that not only a local, but also a systemic strong adaptive immunity was induced in resistant lines. In addition, the presence of high levels of circulating IgA specific both for adult as larvae-derived antigens since the beginning of the infection in resistant animals might suggest a systemic response and an impairment in memory B cells in susceptible lambs. Indeed, memory B cells can be reactivated much more quickly than naïve lymphocytes and might provide a more rapid and lasting protective immunity. However, more experiments are needed to verify this hypothesis.

In agreement with our results, previous works have reported that production of IgA can be an indicator of immune responsiveness to gastrointestinal nematode infection^10,16,22–24^ since it was negatively correlated with faecal EPG^25,26^ and abomasal adult worm count in lambs^27^, and could constitute a marker for resistance selection^12^. The identification of the specific epitopes present in L3 parasites recognized by these IgA antibodies will be key to understand their role in parasite recognition and elimination, as well as to develop prevention and diagnosis strategies.

IgG antibodies specific for adult-derived antigens were also found in high titers in plasma of resistant lambs from the Rylington x Corriedale crossbreed, but not the susceptible or resistant Corriedale lambs. IgG, as IgA, can mediate ADCC by FcγR binding in effector cells. Activating FcγR are expressed on different immune cells such as monocytes, neutrophils or dendritic cells, enhancing phagocytosis, the respiratory burst-induced production of reactive oxygen species or the production of inflammatory cytokines, which enhances the immune response against pathogens. Interestingly, IgG polyclonal antibodies elicited by the nematode *Heligmosomoides plygyrus* infection in mice limit parasite fecundity by low-affinity interactions with parasite antigens although this function seems to be complement- and FcγR-independent^16^. In this case, polyclonal IgG bind and interfere with the function of secreted parasite antigens required for essential functions such as feeding or reproduction^16^. Finally, IgG antibodies can also participate in the promotion of intestinal repair and wound healing during *Heligmosomoides polygyrus bakeri* infection^28^ and interact with mast cells via Fcγ receptors enhancing immunity by the release of pro-inflammatory mediators.

It is worth noting that control animals, non experimental infected, presented a slight increase in specific antibodies in particular days during the experiment. The increase in antibody levels could respond to the presence of a low parasite load in these animals, infected with the pastures that could get contaminated. In fact, experimentally infected and control animals shared the space, and parasite contamination coming from experimental infected animal faeces cannot be excluded. Indeed, susceptible animals showed an average of 100 EPG during the experiment, which could elicit a specific immune response in these animals. Finally, we have to highlight that all animals were previously naturally infected, and a low parasite load can be sufficient to elicit secondary antibody responses.

In addition to the antibody response, differential T cell polarization is associated with parasite resistance and/or susceptibility^8,10,29^, since IgA production is regulated by cellular immunity and Th2 T cell activation^30,31^. We found that PBMC from resistant Corriedale lambs produced higher levels of IL-4 and IL-13 while PBMC from resistant Rylington x Corriedale lambs produced higher levels of IL-5 and TNF*α* than the other lines. IL-4, IL-5 and IL-13 are the hallmark cytokines of type-2 immunity, both at the innate and the adaptive level and have been associated to nematode resistance^8^. Together, IL-4 and IL-13 promote increased contractility of smooth muscle cells in mice^11^, increased permeability of epithelial cells^32^ and elevated goblet cell hyperplasia during nematode infection^33^. The presence of IL-4 in extravascular tissue induces alternative activation of resident tissue macrophages, which function in wound healing and tissue repair^11,32,33^. IL-13 induces epithelial cell repair and mucus production, and together with IL-9, recruits and activates mucosal mast cells. On the other hand, IL-5, apart from triggering eosinophil differentiation, enhances secretion of IgA by B cells^34–36^. Although TNFα is not a typical Th2 cytokine since it mediates pathogenesis in a board range of Th1-mediated diseases, it participates in the regulation of Th2 cell–mediated protection during helminth infection has already been reported^37,38^. In fact, TNF-α is known to regulate expression of a range of cell adhesion molecules on vascular endothelium and leukocytes, participating in the homing of Th2 cells to the site of inflammation^39,40^ or amplification of an existing Th2 response^37,38^. In addition, TNFα plays a critical role in host protection against an intestinal helminth infection^37,38^.

In sheep, a Th2 response characterized by mast cell hyperplasia, eosinophilia, recruitment of IgA/IgE producing cells and the expression of Th2 cytokines, is considered to promote the development of resistance^18,19^. Indeed, previous studies in mice^7,41,42^ and sheep^8^ have highlighted the central role of IL-4 and IL-13 within the abomasal mucosa in the control of gastrointestinal nematodes and maintenance of resistance^6,8,9,29,43^. Furthermore, persistently-infected susceptible animals that carry high parasite loads develop an inflammatory Th1/Th17 response within the abomasum that fails to control infection^6,43^. This indicates that Th2-polarized T cells may play an important role in the maintenance of resistance, when present at the site of infection. Thus, our results complement this research on type-2 cytokines in the mucosa since PBMCs are able to produce high levels of these cytokines upon stimulation by parasite components, suggesting that the Th2 polarization goes far beyond from the site of the infection and that parasite-specific T cells in circulation are polarized towards a Th2 adaptive cell response.

In conclusion, the results presented in this work indicate the induction of a rapid and sustained parasite-specific local and systemic antibody immune response associated to a strong Th2 polarization in resistant sheep lines obtained upon selection by their natural resistance to infection. Although susceptible lambs can produce high levels of IgA antibodies both in saliva and plasma, their antibody response is delayed, which together with an impaired specific-Th2 response, may not contribute to initial parasite elimination.

## Methods

### Ethics statement

The Corriedale lambs come from the divergent lines developed the Uruguayan Wool Secretariat in the CIEDAG research station (Centro de Investigación Doctor Alberto Gallinal). Two groups of 12- to 24-month-old male susceptible and resistant Corriedale lambs (n = 12) were used in this study. A third group was used that exhibited high resistant to gastrointestinal nematodes (Rylington x Corriedale) (n = 8). Animals were housed in worm-free conditions at CIEDAG with water supplied *ad libitum* and fed with a commercial pelleted sheep ration through-out the total experimental period. Animal handling and experiments were carried out in accordance with strict guidelines and regulations from the National Committee on Animal Research (Comisión Nacional de Experimentación Animal, CNEA, http://www.cnea.org.uy/, National Law 18.611, Uruguay). All procedures involving animals were approved by Uruguayan Wool Secretariat’s Committee on Animal Research (CNEA Protocol Number: 461).

### Experimental infection and collection of samples

The animals were drenched on arrival with Startect® 10 mg/ml (Derquantel and 1 mg/ml Abamectin) with the recommended dose (1 ml/5 kg body weight) and remained free of parasites (as determined by fecal egg counts) until experimental parasite inoculation. Resistant and susceptible Corriedale (n = 8) and resistant Rylington x Corriedale (n = 6) lambs were orally inoculated with 7500 infective L3 of *H. contortus* administered in three times consecutively at days 0, 1 and 2. Also, non-infected (control) lambs (n = 4) were maintained under the same conditions of infected animals during the experiment. Animals were bled before the infection (day 0) or after 3, 7, 14, 21, 28 and 35 days post-infection (dpi). Saliva and faecal samples were also obtained at these days. At the end of the experiment animals were treated with Startect® 10 mg/ml.

### Parasitological techniques

*H. contortus* parasites underwent through successive inoculations in sheep and purity was analyzed by microscopy. Parasite egg counts per gram of faeces (EPG) were determined using the modified McMaster technique and hematocrit Mean Corpuscular Volume (MCV) and Mean Platelet Volume (MPV) were determined using the Counter 19 from Weiner lab. Leukocyte total counts were determined in a microscope using a Neubauer Haemocytometer.

Thin smears were prepared on individually labelled microscope slides using one or two drops of blood. Smears were air-dried, fixed in absolute methanol, and stained with Giemsa to analyze leukocyte and lymphocyte counts.

### Parasite antigens

Larvae and adult antigens were prepared as described above in order to detect stage-specific antibody levels. One million 3rd stage (L3) larvae of *H. contortus* were used to prepare somatic antigens of larvae^l^ *H. contortus*. Adult Somatic Antigens were prepared with adult worms of *H. contortus* collected from donor sheep with a primary infection of 7000 L3 *H. contortus*. Larvae or adult worms were exposed to three cycles of 20 min of freeze-thawing (from −80 °C to room temperature) in PBS with a cocktail of protein inhibitors (Sigma-Aldrich, St. Louis, MO), homogenized mechanically and followed by ultrasound disruption at 4 °C. The homogenate was then centrifuged at 10,000 g for 30 min at 4 °C and the supernatant was stored at −80 °C until use. Protein concentration of larval and adult lysates was determined using the Bicinchoninic Acid protein assay procedure.

### Determination of parasite-specific antibodies

The levels of IgA in saliva and IgA and IgG in serum were determined by Enzyme-linked immunosorbent assay (ELISA). Ninety-six-well microtitre plates (Nunc, Roskilde, Denmark) were coated overnight at 4 °C with 1 μg/well of larvae or adult lysates in 50 mM carbonate buffer (pH 9.6). After blocking with 1% gelatine in PBS, three washes with PBS containing 0.1% Tween-20 were performed. Serially diluted sera or saliva samples in buffer (PBS containing 0.1% Tween-20 and 0.5% gelatin) were added to the wells for 1 h at 37 °C. Following three washes, wells were treated 1 h at 37 °C using rabbit anti-sheep IgA or IgG peroxidase-conjugate (Biorad) and *o*-phenylenediamine-H_2_O_2_ was then added as substrate. Plates were read photometrically at 492 nm in an ELISA auto-reader (Labsystems Multiskan MS, Finland). Antibody titres were calculated to be the log10 highest dilution, which gave twice the absorbance of control sheep sera with the minor dilution. Titres are shown as the arithmetic mean ± SEM of the log10 titres.

### PBMC cytokine levels by RT-qPCR

PBMC were isolated by Ficoll-Hypaque density gradient centrifugation (GE Healthcare Bio-Sciences AB), within 8 h after collection in citrate-anticoagulated tubes. Isolated PBMC were resuspended in heat-inactivated foetal bovine serum (FBS, Capricorn) containing 10% dimethylsulfoxide, and cryopreserved in liquid N_2_ until used. After defrosting, PBMC viability and counting were determined manually with trypan blue using a hemacytometer. PBMC (5 × 10^5^ cells/well) were seeded in 24-well culture plates and stimulated in triplicates with adult-derived parasite antigens (25 μg/ml) in 1 ml of RPMI medium containing 100 U/ml penicillin, 100 μg/ml streptomycin, 2 mM L-glutamine and 10% heat-inactivated FBS. After 5 days of incubation, cells were collected in Tri-Reagent (Sigma-Aldrich) and stored at −80 °C until use. RNA was purified according to the manufacturer’s instructions. cDNA was synthesised from 1.0 μg RNA using SensiFAST™ cDNA Synthesis kit (Bioline) in 20 μl final volume. qPCR reactions were performed with SensiFAST™ SYBR (Bioline) using 1 μl template cDNA together with,1.0 μl of each primer at 10 mM and nuclease-free water to a final volume of 10 μl. Reactions were performed on an Illumina real-time thermocyler. Amplification was followed by dissociation curve analysis and results were analyzed using the EcoStudy Software (Illumina). The reactions were performed according to the following settings: 95 °C for 5 min for initial activation, followed by 40 thermal cycles of 30 s at 95 °C, and 30 s at 55 °C (IL-13 and GAPDH) or 60 °C (IL-4, IL-5, TNF*α* and GAPDH). Primers for cytokines were the following *IL-4* Forward: 5′-CAGCATGGAGCTGCCT-3′; *IL-4* Reverse: 5′- ACAGAACAGGTCTTGCTTGC-3′; *IL-5* Forward: 5′-CACTGCTCTCCACGCATCAA-3′; *IL-5* Reverse: 5′-TCATCAAGTTCCCATCACCTATCA-3′; *IL-13* Forward: 5′-AGAACCAGAAGGTGCCGCT-3′; *IL-13* Reverse: 5′-GGTTGAGGCTCCACACCATG-3′; *TNFα* Forward: 5′-CCCGTCTGGACTTGGATCCT-3′; *TNFα* Reverse: 5′-TGCTTTTGGTGCTCATGGTG-3′; *GAPDH* Forward: 5′-TCATAAGTCCCTCCACGATG-3′; *GAPDH* Reverse: 5′-GGTGATGCTGGTGCTGAGTA -3′.

### Statistical analysis

Results were analyzed using GraphPad Prism software (GraphPad Software, San Diego, CA) by two-way ANOVA. Results were considered to be significantly different when p < 0.05.

## Supplementary information

Supplementary Figures

## Data Availability

The authors confirm that the data supporting the findings of this study are available within the article and its Supplementary Materials.
