# Supplementary material for: Resistance to Haemonchus contortus in Corriedale sheep is associated to high parasite-specific IgA titer and a systemic Th2 immune response
Source: Sci Rep. 2019 Dec 20;9:19579. doi: 10.1038/s41598-019-55447-6 (PMC6925110; doi:10.1038/s41598-019-55447-6)
Supplement: Supplementary file 1 — Supplementary Figures [file 41598_2019_55447_MOESM1_ESM.pdf]

Supplementary Figures

Resistance to *Haemonchus contortus* in Corriedale sheep is associated to high parasite-specific IgA titer and a systemic Th2 immune response

Cecilia Escribano[1,2], Anderson Saravia[2], Monique Costa[1], Daniel Castells[3], Gabriel Ciapesoni[4], Franklin Riet-Correa[2] and Teresa Freire[1*]

[1]Grupo de Inmunomodulación y Desarrollo de Vacunas, Departamento de Inmunobiología, Facultad de Medicina, Universidad de La República, Montevideo, Uruguay.
[2]Plataforma de Salud Animal, Instituto Nacional de Investigación Agropecuaria, La Estanzuela, Uruguay.
[3]Secretariado Uruguayo de Lana, Florida, Uruguay.
[4]Programa Carne y Lana. Instituto Nacional de Investigación Agropecuaria, Las Brujas, Uruguay.

Supplementary Figure S1

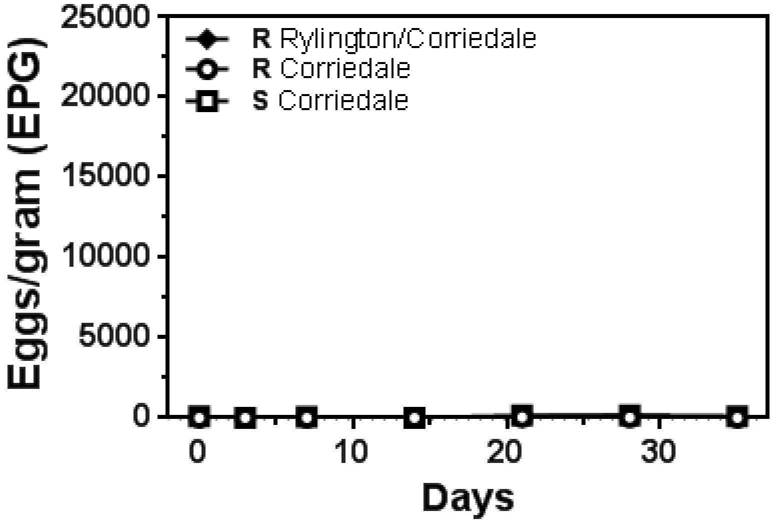

Supplementary Figure S1. EPG from non infected sheep lines. EPG from resistant and susceptible Corriedale and resistant Rylington x Corriedale (n=4) lambs that were not infected with *H. contortus* and remained to be used as control animals during the experiment.

Supplementary Figure S2

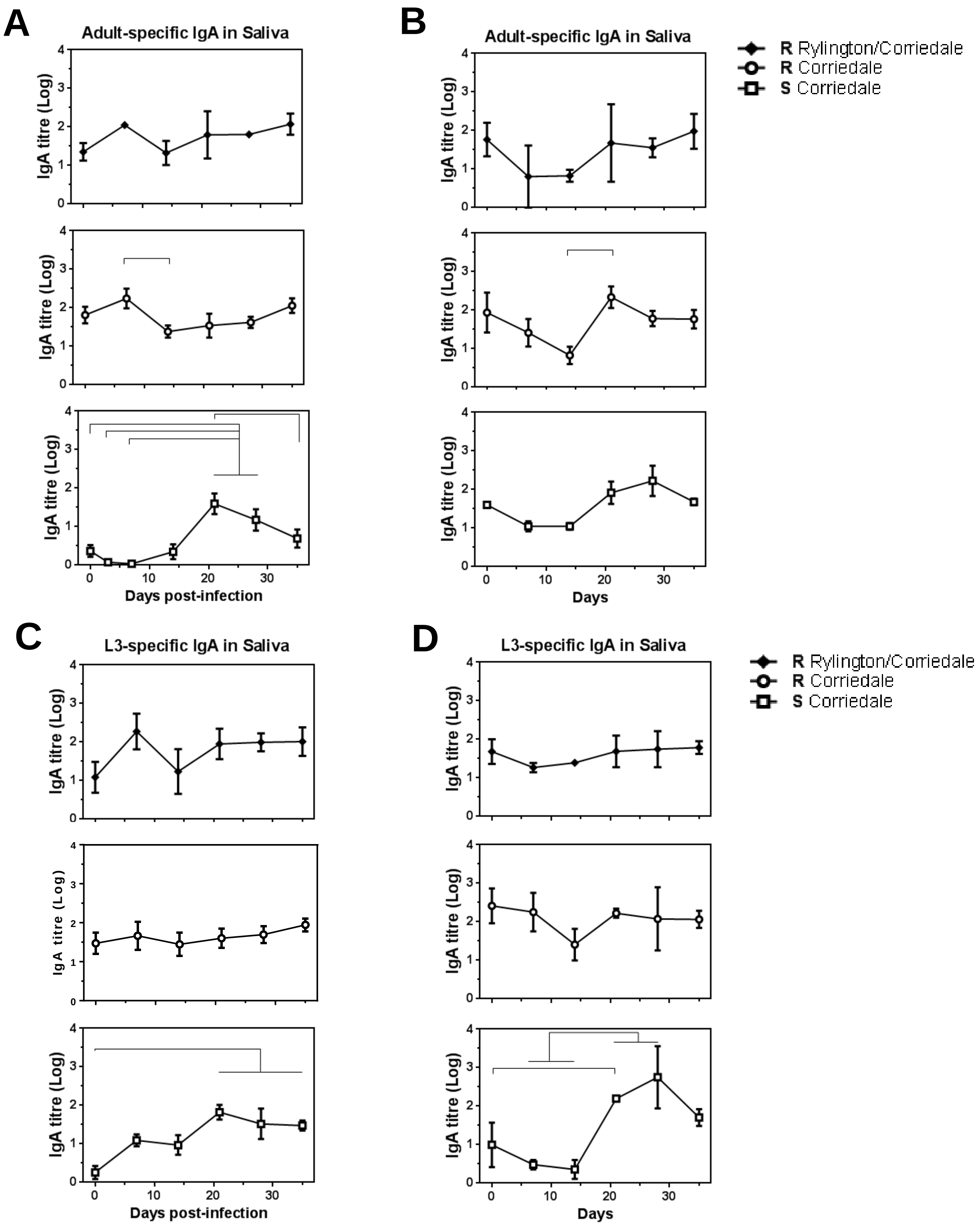

Supplementary Figure S2. Significant differences during the infection for the parasite-specific IgA titers levels in saliva. Saliva samples from infected (A, C) or control (B, D) susceptible and resistant Corriedale and resistant Rylington x Corriedale lambs were incubated with larvae (A, B) or adult (C, D) derived-antigen coated plates. Specific antibodies were detected with anti-sheep IgA HRP-conjugated antibody.

Supplementary Figure S3

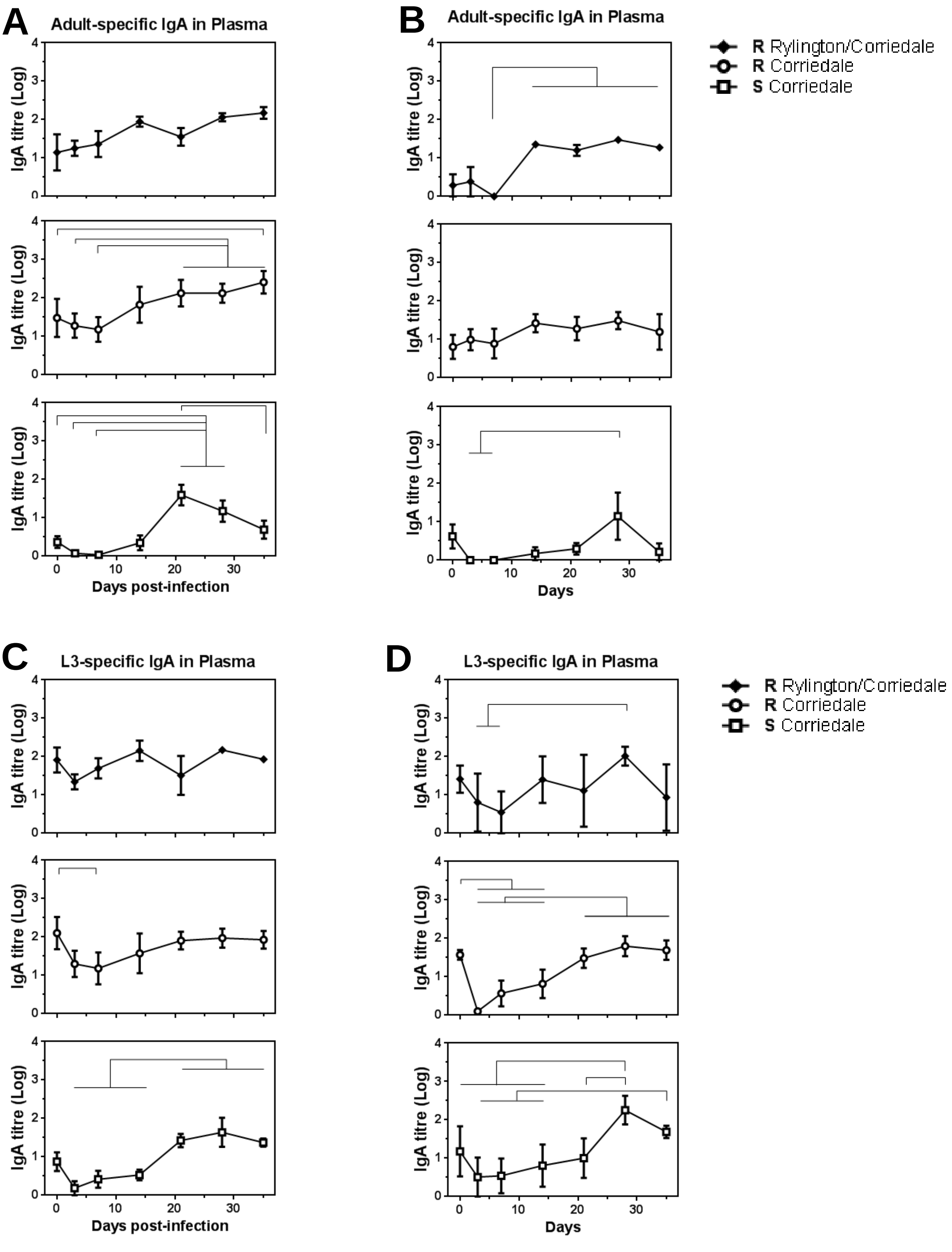

Supplementary Figure S3. Significant differences during the infection for the parasite-specific IgA titers levels in plasma. Saliva samples from infected (A, C) or control (B, D) susceptible and resistant Corriedale and resistant Rylington x Corriedale lambs were incubated with larvae (A, B) or adult (C, D) derived-antigen coated plates. Specific antibodies were detected with anti-sheep IgA HRP-conjugated antibody.

Supplementary Figure S4

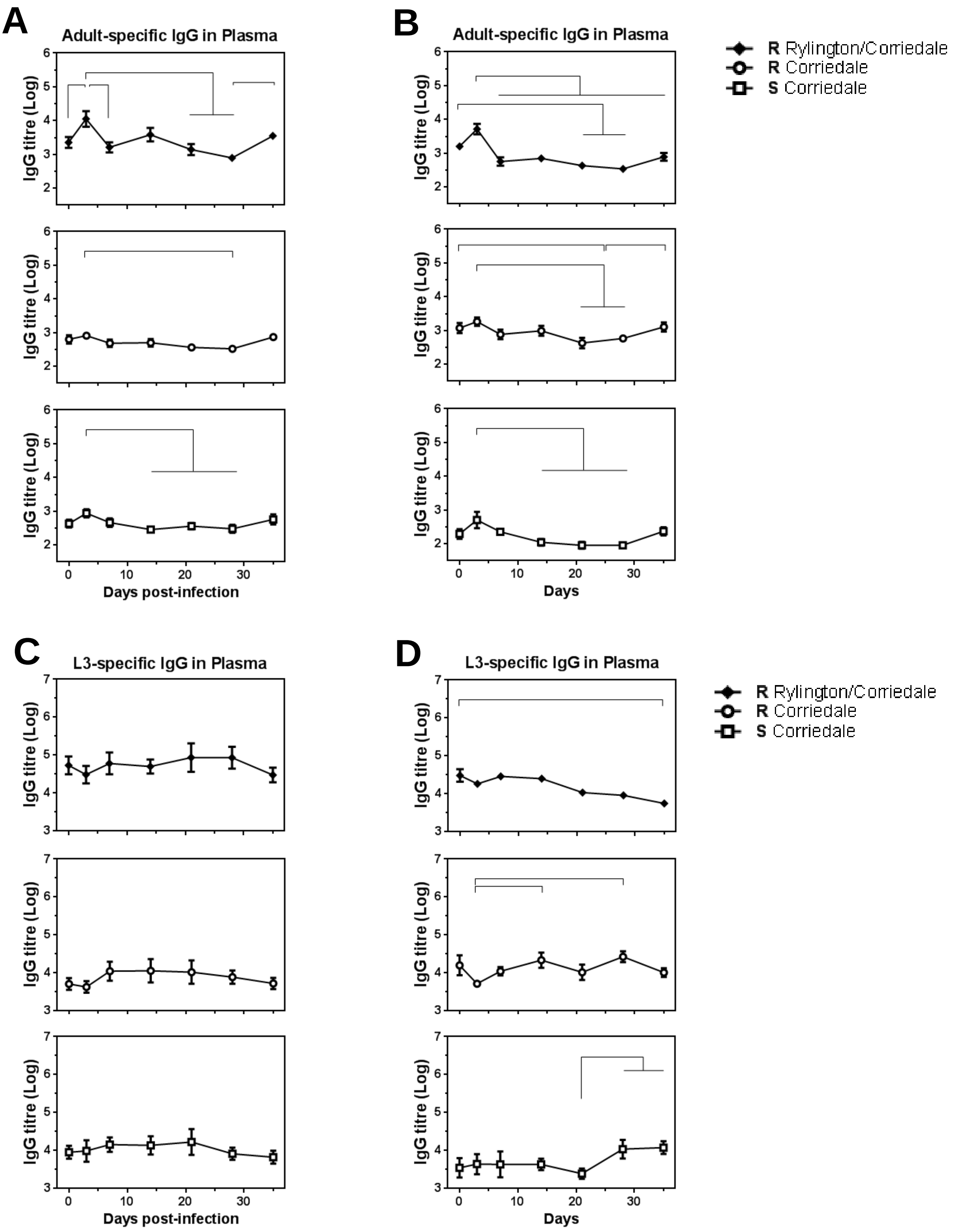

Supplementary Figure S4. Significant differences during the infection for the parasite-specific IgG titers levels in plasma. Saliva samples from infected (A, C) or control (B, D) susceptible and resistant Corriedale and resistant Rylington x Corriedale lambs were incubated with larvae (A, B) or adult (C, D) derived-antigen coated plates. Specific antibodies were detected with anti-sheep IgA HRP-conjugated antibody.

Supplementary Figure S5

Day 35

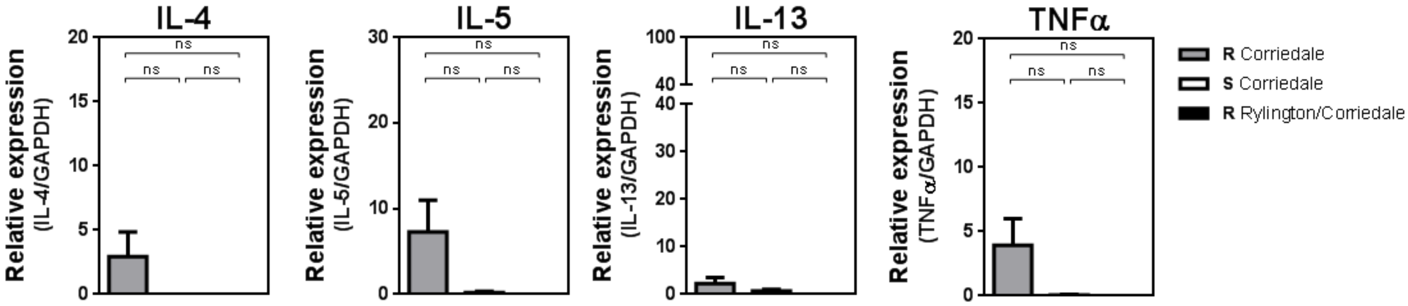

Supplementary Figure S5. Type-2 cytokine levels produced by PBMC from control animals. PBMCs from non infected susceptible and resistant Corriedale and resistant Rylington x Corriedale lambs obtained at 35 days after infection were stimulated with adult-derived parasite antigens for 5 days. Copy DNA from these cells was used as a template to determine cytokine mRNA levels by qRT-PCR. Results are shown as the ratio of mRNA amplification for a specific cytokine and the GAPDH ± SEM.
